# Navigating latency hurdles: an in-depth examination of a cloud-powered GNSS real-time positioning application on mobile devices

**DOI:** 10.1038/s41598-024-65652-7

**Published:** 2024-06-25

**Authors:** Jorge Hernández Olcina, Ana B. Anquela Julián, Ángel E. Martín Furones

**Affiliations:** Department of Cartographic Engineering, Geodesy and Photogrammetry, Universitat Politècnica de València, Camino de Vera s/n, 46022 Valencia, Spain

**Keywords:** Real-time GNSS, Smartphone, Cloud computing, Latency, Android, Engineering, Software

## Abstract

A growing dependence on real-time positioning apps for navigation, safety, and location-based services necessitates a deep understanding of latency challenges within cloud-based Global Navigation Satellite System (GNSS) solutions. This study analyses a GNSS real-time positioning app on smartphones that utilizes cloud computing for positioning data delivery. The study investigates and quantifies diverse latency contributors throughout the system architecture, including GNSS signal acquisition, data transmission, cloud processing, and result dissemination. Controlled experiments and real-world scenarios are employed to assess the influence of network conditions, device capabilities, and cloud server load on overall positioning latency. Findings highlight system bottlenecks and their relative contributions to latency. Additionally, practical recommendations are presented for developers and cloud service providers to mitigate these challenges and guarantee an optimal user experience for real-time positioning applications. This study not only elucidates the complex interplay of factors affecting GNSS app latency, but also paves the way for future advancements in cloud-based positioning solutions, ensuring the accuracy and timeliness critical for safety–critical and emerging applications.

## Introduction

The advent of GNSS technology in smartphones has revolutionized the way we perceive and interact with our surroundings. The ability to accurately determine one’s position has found applications in numerous fields, from navigation and geolocation services to scientific research and emergency response. The Android operating system has made significant strides in GNSS technology. Prior to the release of Android 7 (Nougat) in 2016, only the position–velocity–time (PVT) computed by the GNSS chipsets was available to users, with positioning accuracy typically between 3 and 5 m. However, with the release of Android 7, raw GNSS measurements, including pseudorange, carrier-phase, Doppler shift, and carrier-to-noise density ratio (C/N0) observations, became accessible, paving the way for more precise positioning applications^1^.

The demand for high-precision and reliable positioning systems has pushed the capabilities of receivers to their limits, necessitating innovative strategies to enhance their performance in resource-constrained environments. Efficiently processing GNSS data while grappling with size, energy, and computational constraints is now crucial for propelling positioning and navigation technologies forward. Tackling these challenges will play a pivotal role in shaping the future of GNSS applications and catering to the diverse needs of users across various domains. Therefore, the need to address these challenges efficiently has given rise to the concept of harnessing cloud computing capabilities^2–5^. Cloud computing offers a solution to the hurdles associated with GNSS data handling and processing within receivers. Additionally, this approach addresses the issue of energy consumption, as many GNSS receivers depend on batteries that drain rapidly under increased computational demands^4^. Cloud computing provides a promising path for offloading computationally intensive tasks to remote servers, thereby alleviating the load on local devices and optimizing energy utilization. This change in basic assumptions opens new possibilities for more resource-efficient and sustainable positioning and navigation solutions within the GNSS technology landscape.

While scholarly inquiry into cloud computing’s application in smartphone-based positioning remains limited, emerging research indicates promising advancements in utilizing this technology for geospatial data analysis and processing. Works by^6,7^, advocate for the integration of cloud computing in geospatial data processing. Konstantinos et al.^6^ proposed a client–server architecture tailored for managing geospatial data, which is adaptable to the context explored in this study. In this framework, the client, represented by a smartphone, collects GNSS data and transmits them to the server for processing, determining the smartphone’s position. Similarly, Liu et al.^7^ adopted a comparable methodology, focusing on Real-Time Kinematic (RTK) positioning. Their approach outlines an algorithm for server-side data acquisition and processing, facilitating precise position calculation based on accurate time synchronization. Both studies underscore the potential of cloud computing to optimize geospatial data processing, thus paving the way for more precise and efficient positioning solutions. By harnessing cloud resources, these methodologies address the limitations of client-side capabilities, demonstrating the transformative role of cloud-based methodologies in advancing geospatial applications.

Hence, this study aims to investigate the latency issues encountered in an Android application utilizing GNSS and cloud computing for position calculation. The research focuses on identifying the contributing factors to latency and proposes potential strategies for mitigation. For cloud-based position computation, the RTKLib tool has been selected, inspired by previous studies such as that of Everett^8^, which aimed to optimize RTKLib for measurements conducted with Android smartphones. These studies underscored the need for adjustments to accommodate the suboptimal quality of measurements obtained from mobile platforms. By combining cloud computing with high-performance software featuring intricate algorithms, this approach holds promise for enhancing positioning accuracy while alleviating the computational burden on smartphones.

## Methodology

### App and cloud computing setup

A meticulously crafted Android application has been developed to facilitate real-time GNSS positioning through cloud-based computation. This application serves a dual function by seamlessly integrating offline data logging and online real-time processing, effectively functioning as a GNSS receiver. The application enables direct access to the smartphone’s GNSS sensor via the Android API^9^, allowing for the retrieval of raw GNSS data, including pseudoranges, carrier phases, and Doppler measurements. On the server side, the backend infrastructure is built upon the RTKLib (v2.4.3 b34) calculation engine, module RTKNavi^10^. The communication between the application and server is established through the WebSocket network protocol, chosen for its real-time advantages and unique characteristics^11^.

**Figure 1:**
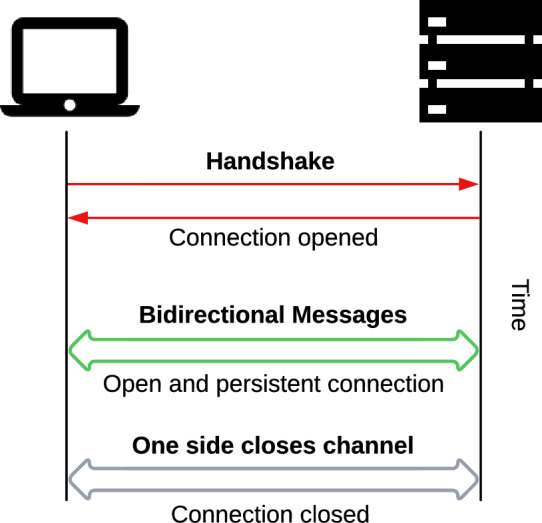
WebSocket connection diagram.

This vital communication link, described in Fig. 1, is widely acknowledged as the most effective method for real-time applications due to its distinctive advantages. WebSockets are favoured in real-time communication for several compelling reasons. Firstly, they boast low latency and support bi-directional communication, ensuring swift and responsive data exchange. Additionally, they are efficient and lightweight, optimizing resource usage without compromising performance. Their ability to maintain continuous connections facilitates seamless real-time updates, enhancing user experience. Moreover, WebSockets offer scalability, accommodating growing demands effortlessly. Their event-driven architecture further enhances responsiveness, enabling applications to react promptly to changes. Finally, their robust security measures safeguard data integrity and confidentiality, making them a reliable choice for diverse communication needs^12^.

**Figure 2:**
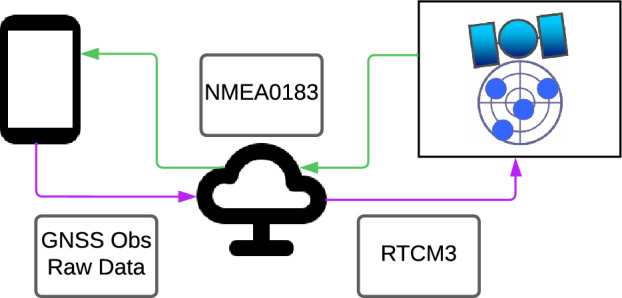
Cloud computing platform workflow.

As illustrated in Fig. 2, the application functions as a GNSS receiver, it captures raw data from the smartphone’s sensor, including satellite Pseudoranges, Carrier phases and Doppler measurements, and initiates the WebSocket connection to the server. This connection prompts the server to prepare for execution and launch an RTKLib instance. Concurrently, bidirectional TCP/IP connections are established to facilitate efficient data exchange. Upon connection establishment, the application sends a “start” message, flagging the server’s readiness for data collection and processing. Once the raw GNSS data are received, the synchronized server conducts a crucial preprocessing step by converting raw GNSS data into the widely used RTCM3 format (Radio Technical Commission for Maritime Services—Version 3), essential for real-time differential GNSS applications^13^.

Converting to RTCM3 format involves a sequence of computations to derive crucial measurements such as Pseudorange, Carrier Phase, Doppler, and temporal values. As outlined by European Global Navigation Satellite System (GSA)^9^, obtaining these measurements directly from the Android API necessitates several steps. First, GPS Time Generation is required, which, in the context of Android 7 or higher, involves utilizing an internal hardware clock and adjusting it to approximate GPS time in nanoseconds, provided the receiver has synchronized with the GNSS reference time. Despite potential delays caused by latency in receiving observations on the server, the position calculation is performed based on the reception time or the time at which the raw GNSS data is received by the smartphone. This ensures that the position calculation is synchronized with the timing of the received data, enabling accurate and timely determination of the user’s position despite any transmission delays. Second, Pseudorange Generation involves computing pseudoranges based on temporal disparities between reception and transmission instances, utilizing essential parameters available from the Android system. Third, Carrier Phase Measurements are obtained as *AccumulatedDeltaRangeMeters* (ADRM) represented in meters. Finally, Doppler Measurements are derived from the *PseudorangeRateMetersPerSecond* parameter, with caution required as it represents an “uncorrected” value, lacking adjustments for receiver and satellite clock frequency discrepancies. A positive “uncorrected” value indicates satellite motion away from the receiver.

Once the previously delineated computations were executed, the transmission of the GNSS raw data to RTKLib was facilitated through the utilisation of the MSM5 (Multiple Signal Messages—Type 5) message from the RTCM3 format, which is designed to carry satellite observations from multiple constellations and frequencies. The converted RTCM3 MSM5 messages, along with necessary navigational information, are then transmitted to RTKLib for precise positioning solution computation. After successful computation, the solution is transmitted back to the server through established TCP/IP connections. The server subsequently forwards the computed solution to the smartphone, which, acting as the end-user interface, stores the solution in plain text files adhering to the National Marine Electronics Association (NMEA0183) format. These files can be readily accessed and interpreted by users for accurate positioning information, effectively demonstrating the real-time capabilities of the system.

In summary, this integrated system exemplifies the effectiveness of a cloud-based approach for real-time GNSS positioning on Android devices. The incorporation of WebSockets enhances communication efficiency, making this architecture a promising solution for applications requiring precise and immediate positioning information.

### Latency analysis

In the examination of temporal intervals encompassing the transmission of messages to the server (Data Transmission Latency, DTL), to the reception of positioning solutions in the application (Data Retrieval Latency, DRL) and the computational duration (Cloud Processing Latency, CPL), a specialized NMEA-type message has been incorporated to facilitate timestamp creation.

Example:

- $TIMEST,2,1,270,124,102,547.235,S*73

The structured composition of this message is delineated as follows:

0. Message ID $TIMEST.
1. Total count of messages of this type within the current cycle (one epoch).
2. Sequential message number.
3. Date information (ddmmyy).
4. UTC Time data.
5. Source designation: A (App) or S (Server).
6. Checksum data, invariably initiated with an asterisk (*).

This meticulously designed message format allows for the generation of timestamps, thereby enabling a comprehensive analysis of diverse processing phases. Given the inherent lack of synchronization between the server and the smartphone clock, attempting synchronization poses the risk of introducing passive latency. Consequently, during real-time executions, timestamps are systematically generated to distinguish between those associated with the application and the server, acknowledging the intrinsic temporal disparities. Additionally, it is imperative to note that this methodology addresses the challenges posed by asynchronous clock systems, ensuring a nuanced examination of temporal intricacies in the data transmission and retrieval process.

Considering these factors, there will be two app timestamps incorporating time data synchronized with the smartphone clock. The first timestamp occurs at the message output stage, coinciding with the provision of raw GNSS data. The second app timestamp is registered upon the server’s return of messages containing position calculation information. This dual timestamp arrangement provides a comprehensive temporal overview, encompassing the duration from message initiation to position computation.

Conversely, the server will generate two timestamps synchronized with the server clock as well. The first timestamp is marked at the instant the server receives the incoming message, while the second timestamp is recorded when the server dispatches the RTKLib output messages. This dual timestamp approach on the server side is instrumental in capturing and assessing the processing time involved in the entire operation.

### Data collection

The evaluation of latency behaviour is undertaken through a series of comprehensive tests designed to ascertain its potential impact on positioning accuracy. Two distinct evaluations have been conducted across varied settings to comprehensively assess the latency effect. The first evaluation involves a static test spanning approximately four hours, at a frequency of 1 Hz, while the second entails two kinematic assessment lasting approximately fifteen minutes, at a frequency of 1 Hz. Both tests adhere to a standardized configuration for hardware and software, described in Tables 1 and 2 respectively, ensuring consistency and comparability across evaluations.

**Table 1:** Hardware specifications.

Device	Android	Psdorange	ADRM	Systems	Network
Google Pixel 7 Pro	14	Yes	Yes	GPS GAL GLO BDS	Static: 4G + Vodafone Kinematic: LTE + Telekom

**Table 2:** RTKLib configuration^10^.

Options	Settings value
Positioning mode	Single
Frequency	L1
Elevation mask	15°
Ionosphere correction	Klobuchar
Troposphere correction	Saastamoinen
Satellite ephemeris/clock	Broadcast
Constellations	GPS, GAL, GLO, BDS

On the one hand, the static test aims to simulate prolonged operational conditions, allowing for a nuanced understanding of latency’s influence over extended durations. In this scenario, particular attention is directed towards analysing the change points of the ephemeris dataset. The primary aim is to discern whether alterations in the navigation message prompt discernible peaks in positional error, potentially attributable to the utilization of outdated ephemeris data resulting from latency in computational processing.

On the other hand, kinematic tests are designed to capture real-time dynamics and rapid fluctuations in positioning accuracy, providing insights into the immediate impact of latency. Two distinct kinematic tests were conducted, one involving pedestrian movement and the other conducted while driving, to analyse contrasting real-world scenarios: low and high speed, respectively. The objective is to assess the effect of latency in both scenarios and evaluate the technology’s feasibility in situations where real-time solutions are critical. These tests entail a comparative analysis between real-time solutions generated by RTKLib and post-processed solutions derived from raw data collected during live executions. For this purpose, the stored raw GNSS data are processed using the RTKPost module, with the data having been previously converted to the RINEX format^14^. This comparative methodology establishes a benchmark, with the post-processed solution serving as a reference for evaluating the computational real-time accuracy. Besides, this framework facilitates the quantification of positional discrepancies induced by latency.

Finally, considering the implementation of timestamp latency messages elucidated in the preceding section, a specialized tool has been designed to parse the output file generated by the application, containing real-time execution results. This tool facilitates the generation of a graphical representation, wherein latency results including Data Transmission, Data Retrieval, and Cloud Processing Latency are graphically depicted for each calculation epoch. Furthermore, the tool provides a comprehensive array of statistical analyses aimed at elucidating the intricacies of latency behaviour throughout the execution process. The graphical representation affords a visual understanding of latency dynamics across successive calculation epochs, thereby enabling the identification of any temporal patterns or anomalies in latency metrics. By delineating the temporal evolution of latency components, such as data transmission and cloud processing, the graphical output offers valuable insights into the performance of the system under varying computational loads and network conditions. This tool serves as a vital instrument for comprehensively assessing and monitoring latency dynamics throughout the execution process, thereby empowering stakeholders to optimize system performance and mitigate potential bottlenecks in real-time data processing workflows.

## Results and discussion

### Static test

In the conducted static test, the hardware specified in Table 1 was deployed at a fixed outdoor location, for an approximate duration of four hours. The objective was to investigate the impact of latency on error occurrences during transitions between navigation messages. Table 2 outlines the RTKLib configuration employed for position determination.

**Figure 3:**
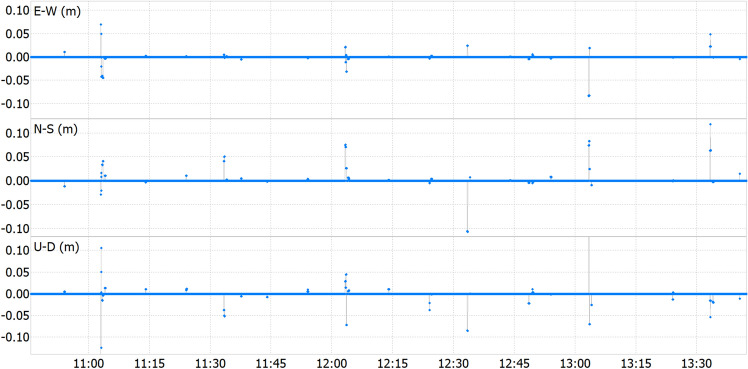
Real-time versus post-processing static test ENU position differences.

Given that satellite positions are computed using orbital propagation based on the Keplerian parameters of the navigation messages over time, concerns arose regarding potential discrepancies resulting from latency-induced delays during ephemeris updates. Real-time results were leveraged to scrutinize positional variations over the test duration. Figure 3 depicts the comparative analysis, revealing that latency fluctuations did not induce significant deviations in any of the three positional components when comparing the solution in real-time with the solution in post-processing. In addition, Table 3 provides a statistical overview of the disparities, or differences, observed between real-time processing and post-processing methods. The data illustrates that, while maximum peaks exceeding 10 cm may occur, the mean and standard deviation indicate minimal divergence between the results obtained through real-time and post-processing. Despite the expectation of no disparities between the two methods in theory, certain factors can contribute to differences.

- *Satellite geometry and signal quality* Differences in satellite geometry and signal quality between real-time and post-processed data can lead to discrepancies in positioning accuracy^15^.
- *Atmospheric conditions* Variations in atmospheric conditions, such as ionospheric disturbances or tropospheric delays, can affect GNSS signals differently in real-time versus post-processing scenarios^16^.
- *Receiver clock errors* Inconsistencies in receiver clock parameters for code and carrier phase observations can impact the accuracy of GNSS positioning, especially when using a common clock parameter for both types of measurements^15^.
- *Data processing algorithms* The algorithms used for real-time processing and post-processing may differ, leading to variations in how data is handled and corrected, influencing the final positioning results^15^.
- *Multi-constellation solutions* Utilizing multi-constellation solutions, like GPS combined with Galileo, can significantly improve accuracy in post-processing compared to real-time solutions due to enhanced signal availability and redundancy^15^.

**Table 3:** Static test statistics on real-time versus post-processing solution disparities.

	dE (cm)	dN (cm)	dU (cm)
Maximum (abs)	8.3	11.8	17.57
Minimum (abs)	0.0	0.0	0.0
Standard deviation	0.3	0.5	0.7
Mean	0.0	0.0	0.0
Median	0.0	0.0	0.0

Given the minimal disparities observed, attention can be focused on the issue of latency, the communication methodology between the application and server was pivotal in mitigating latency effects, optimizing network capabilities, and yielding latency results well within acceptable thresholds, thereby safeguarding against errors in static positioning. While acknowledging the multifactorial nature of latency, the findings affirm the viability of the communication approach for static position calculations. Consequently, it is concluded that the methodology is promising for static positioning applications.

### Kinematic test: *pedestrian*

To assess the impact of latency in kinematic scenarios, a real-world walking test was conducted. Utilizing the hardware and configuration detailed in Table 1, and the RTKLib setup outlined in Table 2, a 15-min test was executed under consistent walking conditions. The test route followed an oval trajectory encompassing areas with varied visibility, including wooded regions known to potentially affect positioning solutions.

**Figure 4:**
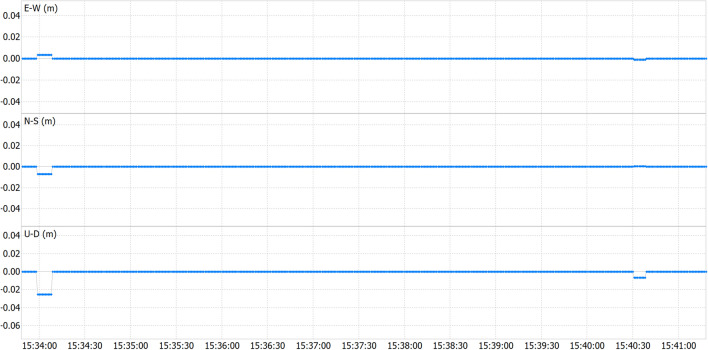
Real-time versus post-processing pedestrian test position differences.

Comparative analysis was performed between real-time positioning results and post-processed data obtained from the same route. As highlighted in preceding sections, the application not only captures real-time RTKLib outputs but also archives raw sensor data for potential post-processing. Figure 4 presents the difference between both solutions, revealing negligible positional disparities, often below one millimetre, highlighting the magnitude of the differences between the two methods.

**Figure 5:**
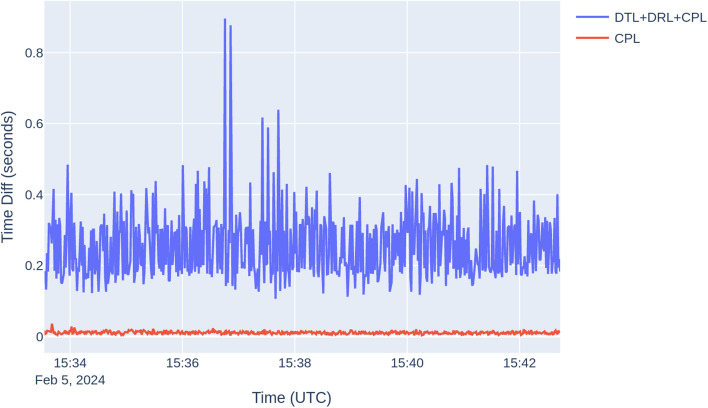
Pedestrian test latency plot.

Figure 5 illustrates the latency metrics derived from the test. The blue line represents the duration between raw data transmission to the server and reception of positioning results, while the red line denotes server-side processing time, encompassing the duration from data receipt to result dispatch. Additionally, Table 4 provides a concise summary of latency statistics extracted from the graph. The graph and table illustrate the latency distribution within the whole GNSS cloud computing platform. They depict that processing time has the least impact, with most of the latency attributed to transmission time (DTL) and reception time (DRL). This insight underscores the significance of optimizing these phases to enhance overall system performance and user experience.

**Table 4:** Static test statistics on real-time versus post-processing solution disparities.

	DTL + DRL + CPL (s)	CPL (s)
Maximum	0.8960	0.0360
Minimum	0.1060	0.0030
Standard deviation	0.0947	0.0039
Mean	0.2556	0.0105
Median	0.2240	0.0100

Analysing the results from Table 5, minimal differences are once again evident between real-time and post-processing methods, with a maximum peak difference of only 2 cm vertically. These slight disparities may be attributed to numerous factors outlined in the static test. However, they further validate the methodology and affirm that it does not introduce significant errors attributable to latency.

**Table 5:** Pedestrian test statistics on real-time versus post-processing solution disparities.

	dE (cm)	dN (cm)	dU (cm)
Maximum (abs)	0.3	0.7	2.5
Minimum (abs)	0.0	0.0	0.0
Standard deviation	0.0	0.0	0.3
Mean	0.0	0.0	0.0
Median	0.0	0.0	0.0

Furthermore, considering the nature of the kinematic test, it is important to acknowledge that due to latency, positions can be displaced depending on the velocity of movement. Assuming an average pedestrian speed of 10 km/h and an average latency of 0.2556 s, the positional variation due to this speed would be approximately 0.7 m. Given the utilization of smartphone-based positioning, it can be inferred that the employed methodology consistently delivers acceptable results regarding latency-induced positional discrepancies.

### Kinematic test: *car*

To assess the potential negative impact of latency in practical scenarios, a car-based test was conducted, covering approximately 15 km along a highway at an average speed of 120 km/h. Consistent with previous tests, the same hardware and configuration were used, as detailed in Tables 1 and 2. The comparative methodology remained consistent, wherein real-time positioning data was juxtaposed with post-processed results.

**Figure 6:**
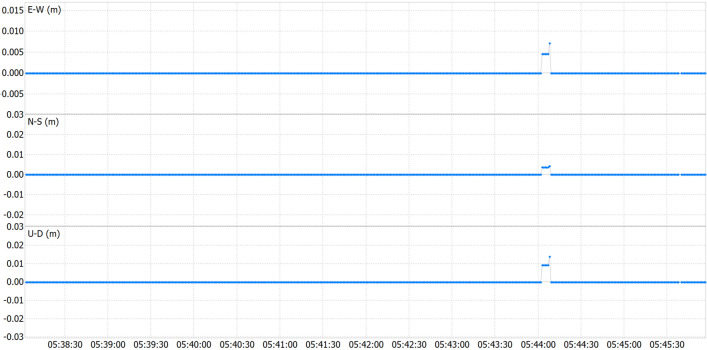
Real-time versus post-processing car test position differences.

**Figure 7:**
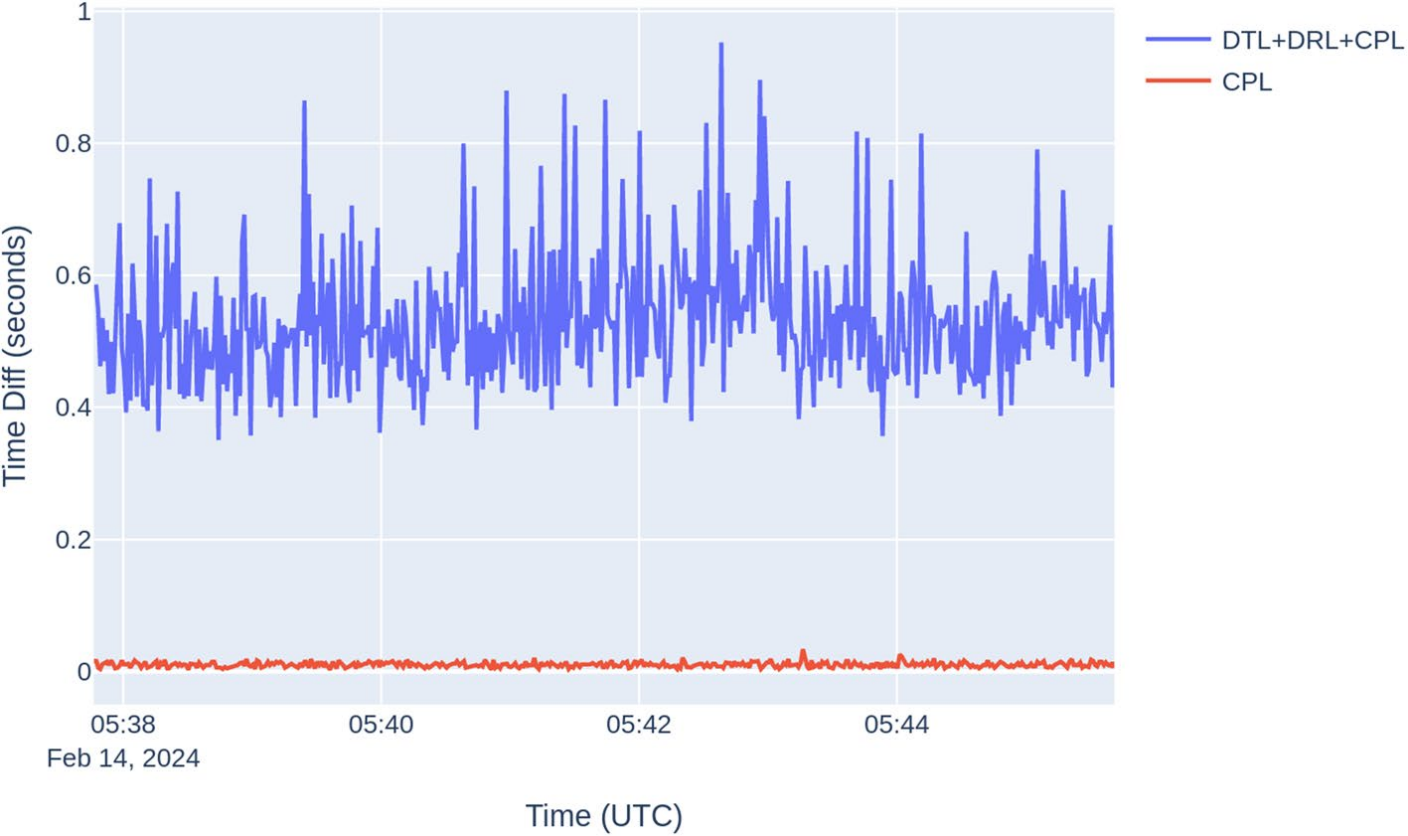
Car test latency plot.

As depicted in Fig. 6, positional variations between real-time and post-processing were minimal, echoing findings from prior tests. Figure 7 illustrates latency results like those observed in previous tests, showing higher DTL, DRL, and CPL. Once more, the data highlights the minimal contribution of CPL to the total latency, with the bulk of the latency primarily stemming from DTL and DRL. This underscores the critical importance of addressing issues related to data transmission and reception to effectively reduce overall latency and improve system efficiency. Furthermore, Table 6 presents latency statistics closely resembling those from preceding experiments. This test has revealed new latency results, which in this instance are higher compared to the previous test, despite it is using the same network. Several factors could contribute to this discrepancy:

- *Network congestion* Variations in network traffic can influence the duration of data transmission between the mobile device and the server. During peak periods of congestion, packets may encounter delays as they navigate through the network^17^.
- *Network latency* The physical distance between the mobile device and the server can introduce latency as data packets travel across the network infrastructure. Additionally, inefficiencies in routing or congestion along the network path can further contribute to fluctuating latency^17^.
- *Serialization and deserialization* Encoding and decoding data sent over WebSockets. The choice of data format (JSON) and serialization library can impact performance^17^.
- *Server processing time* While the graph in Fig. 7 illustrates constant processing time on the server, it is important to consider potential variations due to factors like resource competition, software optimizations, or background tasks running on the server^18^.
- *WebSocket performance* Despite offering a persistent, low-latency connection between the client (smartphone) and the server, WebSocket performance can still be influenced by factors such as network conditions and server responsiveness. Variability in WebSocket performance may contribute to latency fluctuations^12^.
- *Device performance* The performance of the smartphone itself can impact app latency. Factors such as CPU usage, available memory, and background processes on the device can affect the speed of data transmission and reception^18^.

**Table 6:** Static test statistics on real-time versus post-processing solution disparities.

	DTL + DRL + CPL (s)	CPL (s)
Maximum	0.9530	0.0340
Minimum	0.3510	0.0030
Standard deviation	0.0965	0.0038
Mean	0.5313	0.0105
Median	0.5210	0.0100

Considering an average latency of 0.5313 s at a speed of 120 km/h, the resulting positional variation attributed to latency averages around 17 m. At these speeds, the positional variation becomes significantly greater. Therefore, in scenarios where latency is higher, it can result in a substantial displacement of the vehicle’s position by the time the last calculated position is received. This implies that, depending on the application, such discrepancies could be critical.

Once more, Table 7 presents statistics comparing the three position components between real-time and post-processing methods. As observed in previous cases, although occasional peaks of a few centimetres may occur, the disparity in position between real-time and post-processing remains minor. This reaffirms the validity of the methodology even under high-speed conditions.

**Table 7:** Car test statistics on real-time versus post-processing solution disparities.

	dE (cm)	dN (cm)	dU (cm)
Maximum (abs)	0.7	0.4	1.3
Minimum (abs)	0.0	0.0	0.0
Standard deviation	0.1	0.0	0.1
Mean	0.0	0.0	0.0
Median	0.0	0.0	0.0

As a result, this test highlights that at high speeds, even slight increases in latency values can lead to significant positional variations. This suggests that the methodology may not be suitable for applications where security is paramount, despite yielding results. However, it also underscores the potential for further research aimed at reducing latency within the same methodology, thereby opening avenues for improvement and innovation.

**Figure 8:**
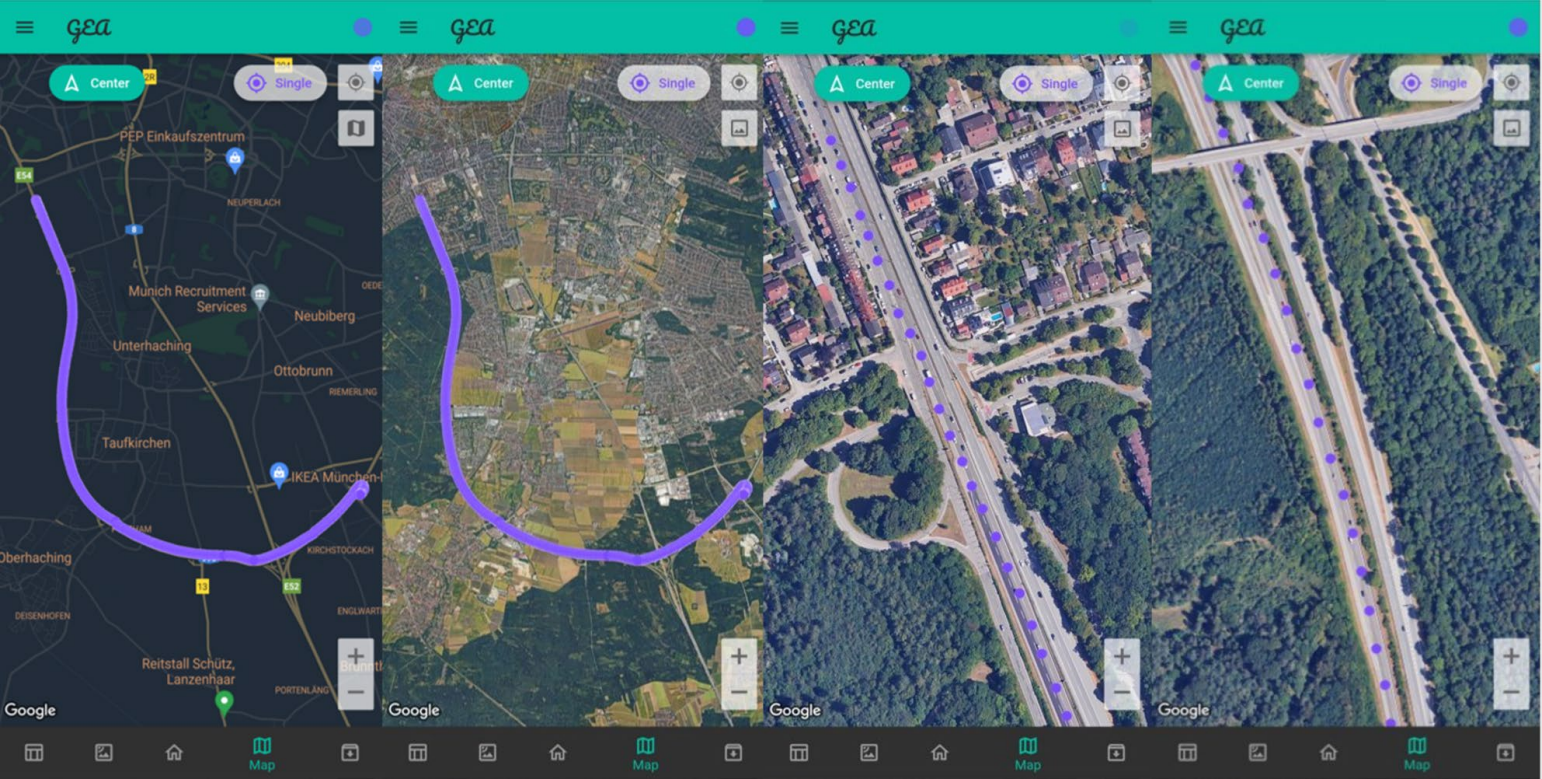
Car test real-time solution snapshots. Maps generated using GEA application version 2.1.0 (https://github.com/jorgeho1995/geagnss/tree/main/releases/GEA-v2.1.0).

Figure 8 offers a comprehensive visual overview, featuring a sequence of screenshots extracted from the application, depicting the test route conducted on a highway by car. These displayed screenshots furnish detailed insights into the performance of the positioning solution. While the primary intent of these findings is not to assess the accuracy of the solution, given its susceptibility to inherent displacement due to speed, they serve to highlight the continuous nature of the solution. The trajectory exhibits smooth transitions without abrupt deviations.

Moreover, the minimal presence of significant outliers attests to the robustness of the employed positioning algorithm. Notably, the solution’s accuracy is commendable, as manifested by the consistent alignment of the route with the actual path traversed, notwithstanding the displacement induced by speed and latency. In summary, the visual representation delineated in Fig. 8 underscores the efficacy and dependability of the application in producing precise positioning solutions under dynamic scenarios, such as vehicular travel on roads. However, it is worth noting, as previously mentioned, that movement at elevated speeds can induce positional shifts attributable to latency.

### Addressing latency concerns and the viability of cloud computing in GNSS applications

The feasibility and practicality of employing cloud computing in applications have been subject to scrutiny within the scientific community. This scrutiny arises from concerns regarding latency, application scope, and the rationale behind such technological pursuits. In response to these concerns, this section aims to elucidate the rationale behind this investigation and shed light on the viability of cloud powered GNSS applications.

This research is driven by mounting concerns over latency challenges in cloud powered GNSS applications, particularly in contexts where real-time positioning is vital. Latency poses significant hurdles in scenarios necessitating precise and timely positioning. Such concerns have spurred thorough investigation into the impact of latency in practical, real-world settings.

This study explored how latency affects a cloud powered GNSS real-time positioning application, focusing specifically on mobile devices. Through thorough experimentation and analysis, it was found that the impact of latency varies depending on the device’s speed. At lower speeds, latency had minimal effect on positioning accuracy. However, as the device’s velocity increased, latency-induced position variations became more significant, emphasizing the importance of effective latency management in high-speed applications.

### Leveraging cloud computing advantages, target applications and future directions

Cloud computing plays a crucial role in addressing challenges related to energy consumption and limited computational capabilities in user devices. By harnessing the computational resources and scalability provided by the cloud, real-time positioning systems can effectively reduce battery consumption issues while improving accuracy and reliability. Additionally, the inherent flexibility of cloud architectures enables the integration of advanced algorithms and techniques, such as Kalman filters, prediction algorithms, and ionospheric monitoring. This integration enhances the functionality and effectiveness of GNSS applications across various domains.

The utility of cloud powered GNSS applications spans a wide spectrum of domains, encompassing navigation, geolocation services, precision agriculture, disaster management, and beyond. By harnessing the power of cloud computing, stakeholders can unlock new possibilities in location-based services, environmental monitoring, and spatial data analysis. Moving forward, further research efforts should focus on optimizing cloud based GNSS solutions, addressing security and privacy concerns, and exploring innovative applications to realize the full potential of this transformative technology.

## Conclusion

This study represents a comprehensive exploration into the nuanced latency challenges inherent in a real-time cloud based GNSS positioning app for smartphones. This app sets in motion an interactive operation by connecting to a specialized server through a WebSocket, which efficiently facilitates the transfer and coordination of data. The server, functioning as a central coordinator, triggers the RTKLib software to perform precise positioning calculations and establishes necessary links for data interchange. Specifically, raw GNSS data undergoes conversion into RTCM3 format, acting as a conduit for further analysis. Following this, RTKLib extracts vital measurements, and the outcomes are relayed to the program for secure retention. The setup underscores the integration of mobile technology, instantaneous communication, cloud-based computing, sophisticated processing, and cloud-driven storage, highlighting its capability to propel GNSS-based positioning applications across various scientific domains.

Through the development and analysis of a bespoke real-time GNSS positioning application leveraging cloud computing for data delivery, a thorough investigation was conducted into the numerous factors contributing to latency across the system.

The research meticulously examined the latency landscape, encompassing critical aspects such as GNSS signal acquisition, data transmission, cloud processing, and the dissemination of results. Utilizing a combination of controlled experiments and real-world scenarios, the study systematically evaluated the impact of network conditions, device capabilities, and cloud server load on overall positioning latency, providing invaluable insights into the intricate interplay of these factors.

By identifying and analysing system bottlenecks, the study offers a nuanced understanding of their relative contributions to latency, thereby providing a roadmap for addressing these challenges. Furthermore, practical recommendations were formulated for developers and cloud service providers to mitigate latency issues and optimize user experience in real-time positioning applications.

The study demonstrates that the precision between the real-time solution and post-processing remains unaffected by latency times. However, the accuracy of the solution in real kinematic scenarios is contingent upon both the velocity at which the smartphone moves and the total processing latency. Based on the experiments conducted, this total latency can range from an average of 0.25 s for users walking to 0.5 s for users traveling at a speed of 120 km/h.

The significance of this research extends beyond its immediate findings, as it lays the groundwork for future advancements in the field. By implementing the recommendations proposed in this study, stakeholders can enhance the accuracy, timeliness, and reliability of real-time positioning systems based on cloud computing, meeting the stringent requirements of safety–critical and emerging applications reliant on instantaneous positioning data.

This study serves as a contribution to the ongoing discourse surrounding real-time GNSS positioning applications, offering actionable insights that have the potential to drive innovation and improve the functionality of such systems in diverse settings and applications.

## Data Availability

The datasets generated and/or analysed during the current study are available in the GitHub repository. https://github.com/jorgeho1995/geagnss.

## Abbreviations

GNSS: Global Navigation Satellite Systems
PVT: Position–velocity–time
C/N0: Carrier-to-noise ratio
RTK: Real-Time Kinematic
TCP/IP: Transmission Control Protocol/Internet Protocol
API: Application Programming Interface
RTCM3: Radio Technical Commission for Maritime Services—Version 3
NMEA: National Marine Electronics Association
SSL/TLS: Secure Sockets Layer/Transport Layer Security
UTC: Universal Time Coordinated

## References

[1] Zangenehnejad F, Gao Y (2021). GNSS smartphones positioning: Advances, challenges, opportunities, and future perspectives. Satell. Navig..

[2] Favenza, A., Rossi, C., Pasin, M. & Dominici, F. A cloud-based approach to GNSS augmentation for navigation services. In *Proceedings of the 7th International Conference on Utility and Cloud Computing* (2014).

[3] García-Molina, J. A. & Parro, J. M. Cloud-based GNSS processing of distributed receivers of opportunity: Techniques, applications and data-collection strategies. In *6th International Colloquium on Scientific and Fundamental Aspects of GNSS/Galileo* (2017).

[4] Lucas-Sabola, V., Seco-Granados, G., López-Salcedo, J. A., García-Molina, J. A. & Crisci, M. Cloud GNSS receivers: New advanced applications made possible. In *2016 International Conference on Localization and GNSS (ICL-GNSS)*. ENC (IEEE, 2018). 10.1109/ICL-GNSS.2016.7533852

[5] Lucas-Sabola, V., Seco-Granados, G., López-Salcedo, J. A., García-Molina, J. A. & Hein, G. W. (2018). GNSS IoT positioning: From conventional sensors to a cloud-based solution. Inside GNSS. *Inside GNS*S. https://insidegnss.com/gnss-iot-positioning-from-conventional-sensors-to-a-cloud-based-solution/. Accessed 31 July 2023.

[6] Konstantinos E, Konstantinos N, Stathis M, Constantine P (2013). Geospatial services in the cloud. Comput. Geosci..

[7] Liu X, Ribot MÁ, Gusi-Amigó A, Rovira-Garcia A, Sanz J, Closas P (2021). Cloud-based single-frequency snapshot RTK positioning. Sensors.

[8] Everett T, Taylor T, Lee DK, Akos DM (2022). Optimizing the use of RTKLIB for smartphone-based GNSS measurements. Sensors.

[9] European Global Navigation Satellite System (GSA) (2016). Using GNSS raw measurements on android devices.

[10] RTKLib. (2013). RTKLib ver. 2.4.2 Manual. https://www.rtklib.com/prog/manual_2.4.2.pdf. Accessed 04 Sept 2023.

[11] Olcina JH, Julián ABA, Furones ÁEM (2023). Treatment and analysis of the GNSS signal from smartphones and its applicability to urban mobility. Environ. Sci. Proc..

[12] WebSockets. (2024). WebSockets Standard. https://websockets.spec.whatwg.org. Accessed 17 Feb 2024.

[13] Radio Technical Commission for Maritime Services (RTCM). RTCM Standard 10403.2. Differential GNSS (Global Navigation Satellite System) Services—Version 3. *RTCM Special Commitee No. 104*, Arlington, Virginia. RTCM Paper 104–2013-SC104-STD (2013).

[14] Hernández Olcina J, Anquela Julián AB, Martín Furones ÁE (2024). Python toolbox for android GNSS raw data to RINEX conversion. GPS Solut..

[15] Wang L, Li Z, Wang N (2021). Real-time GNSS precise point positioning for low-cost smart devices. GPS Solut..

[16] Tondaś D, Kapłon J, Rohm W (2020). Ultra-fast near real-time estimation of troposphere parameters and coordinates from GPS data. Measurement.

[17] Quezada-Gaibor D, Torres-Sospedra J, Nurmi J, Koucheryavy Y, Huerta J (2022). Cloud platforms for context-adaptive positioning and localisation in GNSS-denied scenarios—A systematic review. Sensors..

[18] Allouch A, Cheikhrouhou O, Koubâa A, Toumi K, Khalgui M, Nguyen GT (2021). UTM-chain: Blockchain-based secure unmanned traffic management for internet of drones. Sensors..

